# IL-4Rα-Independent Expression of Mannose Receptor and Ym1 by Macrophages Depends on their IL-10 Responsiveness

**DOI:** 10.1371/journal.pntd.0000689

**Published:** 2010-05-18

**Authors:** Benjamin G. Dewals, Reece G. Marillier, Jennifer C. Hoving, Mosiuoa Leeto, Anita Schwegmann, Frank Brombacher

**Affiliations:** 1 International Centre for Genetic Engineering and Biotechnology (ICGEB), University of Cape Town, Cape Town, South Africa; 2 Division of Immunology, Institute of Infectious Disease and Molecular Medicine (IIDMM), University of Cape Town, Cape Town, South Africa; University of Edinburgh, United Kingdom

## Abstract

IL-4Rα-dependent responses are essential for granuloma formation and host survival during acute schistosomiasis. Previously, we demonstrated that mice deficient for macrophage-specific IL-4Rα (*LysM^cre^Il4ra^−/lox^*) developed increased hepatotoxicity and gut inflammation; whereas inflammation was restricted to the liver of mice lacking T cell-specific IL-4Rα expression (*iLck^cre^Il4ra^−/lox^*). In the study presented here we further investigated their role in liver granulomatous inflammation. Frequencies and numbers of macrophage, lymphocyte or granulocyte populations, as well as Th1/Th2 cytokine responses were similar in *Schistosoma mansoni*-infected *LysM^cre^Il4ra^−/lox^* liver granulomas, when compared to *Il4ra^−/lox^* control mice. In contrast, a shift to Th1 responses with high IFN-γ and low IL-4, IL-10 and IL-13 was observed in the severely disrupted granulomas of *iLck^cre^Il4ra^−/lox^* and *Il4ra^−/−^* mice. As expected, alternative macrophage activation was reduced in both *LysM^cre^Il4ra^−/lox^* and *iLck^cre^Il4ra^−/lox^* granulomas with low arginase 1 and heightened nitric oxide synthase RNA expression in granuloma macrophages of both mouse strains. Interestingly, a discrete subpopulation of SSC^high^CD11b^+^I-A/I-E^high^CD204^+^ macrophages retained expression of mannose receptor (MMR) and Ym1 in *LysM^cre^Il4ra^−/lox^* but not in *iLck^cre^Il4ra^−/lox^* granulomas. While aaMφ were in close proximity to the parasite eggs in *Il4ra^−/lox^* control mice, MMR^+^Ym1^+^ macrophages in *LysM^cre^Il4ra^−/lox^* mice were restricted to the periphery of the granuloma, indicating that they might have different functions. *In vivo* IL-10 neutralisation resulted in the disappearance of MMR^+^Ym1^+^ macrophages in *LysM^cre^Il4ra^−/lox^* mice. Together, these results show that IL-4Rα-responsive T cells are essential to drive alternative macrophage activation and to control granulomatous inflammation in the liver. The data further suggest that in the absence of macrophage-specific IL-4Rα signalling, IL-10 is able to drive mannose receptor- and Ym1-positive macrophages, associated with control of hepatic granulomatous inflammation.

## Author Summary

Schistosomiasis is a tropical disease caused by one of the species of the parasitic worm *Schistosoma* which infects over 200 million people worldwide. Signalling via the IL-4 receptor alpha (IL-4Rα), which is the common receptor chain for the ligands IL-4 and IL-13, is essential for inducing protective Type 2 immune response and granuloma formation in response to the parasite eggs. In experimental *Schistosoma mansoni* infection and egg-induced inflammation studies with cell type-specific IL-4Rα deficient mice, the role of IL-4Rα-activated alternative macrophages (aaMφ) and IL-4Rα-responsive T cells was investigated with focus on the control of hepatic inflammation and granuloma formation. Interestingly, aaMφ were not essential for the cellular composition or the Th1/Th2 cytokine profile in liver granulomas. In contrast, IL-4Rα-dependent T cell responses were important for predominant Th2 and IL-10 responses, as well as the presence of aaMφ in the granulomas, avoiding major disruption in the granuloma cell composition. Moreover, a macrophage subpopulation was identified and those cells expressed the two aaMφ markers, mannose receptor- and Ym1 in an IL-4Rα-independent but IL-10-dependent manner. These cells might be involved in the control of inflammation.

## Introduction

Schistosomiasis is a severe parasitic disease with more than 200 million people infected worldwide with an estimated 280,000 deaths per annum in sub-Saharan Africa alone [1], [2]. In the murine model, mice infected with *Schistosoma mansoni* develop a severe liver pathology with granulomatous inflammatory responses directed towards the parasite eggs. During chronic infections, Th2-type inflammation in the liver results in fibrosis, which leads to portal hypertension, bleeding from collateral vessels and ultimately death [3], [4]. At peak egg excretion, Th2-mediated granuloma formation appears to be indispensable for host protection and control of egg-induced inflammation.

Previous studies have demonstrated signalling *via* interleukin 4 receptor α-chain (IL-4Rα) to be essential for granuloma formation and host survival [5], [6], [7], [8], [9]. The cellular contributions of IL-4Rα to the mechanisms conferring protection to the host can be dissected using mice with IL-4Rα expression disrupted on specific cell types. Infection of macrophage/neutrophil-specific (*LysM^cre^Il4ra^−/lox^*) and T cell-specific (*iLck^cre^Il4ra^−/lox^*) IL-4Rα-deficient mice showed these mice to have high mortality during acute schistosomiasis irrespective of granuloma formation [6], [10]. Eight weeks after infection, the absence of IL-4Rα-dependent T cell responses resulted in severe hepatotoxicity while an absence of IL-4Rα-activated macrophages was responsible for the development of severe inflammation and damage in both intestine and liver, with a subsequent increase in serum lipopolysaccharide (LPS) levels and aspartate transaminase levels in the serum, respectively [6]. Though the mechanism(s) explaining the high mortality rates observed in *LysM^cre^Il4ra^−/lox^* mice is(are) not fully understood, the observed increased Th1/type1 in presence of normal Th2/type 2 cytokine and antibody systemic responses could be detrimental for the host survival [6].

Tissue macrophages were recently classified into three major categories based on their functions [11]. “Classical” macrophage activation occurring during Th1 immune responses, essentially *via* IFN-γ signalling is involved in cellular immunity against intracellular pathogens *via* the production of pro-inflammatory molecules and nitric oxide (NO). Macrophages can also develop a “deactivated” phenotype in association with IL-10 and TGF-β signalling. “Alternative” macrophage activation occurs *via* IL-4 and IL-13 signalling through their heterodimeric IL-4R during Th2 immune responses [11], [12], [13]. Alternative activation of macrophages results in the downstream activation of various molecules/markers among which arginase 1 (Arg-1) has been considered to be decisive for their functional activities [11]. Earlier studies suggested that alternatively-activated macrophages (aaMφ) may be important regulators of wound healing *via* arginase-1 activation. Arg-1 catalyses L-arginine to produce polyamines and proline, an important factor for collagen deposition [14], [15], [16]. More recent studies in *LysM^cre^Il4ra^−/lox^* mice showed that aaMφ are involved in immuno-modulation, -suppression or -pathology in infectious diseases such as schistosomiasis, cutaneous leishmaniasis and cryptococcosis, as well as in non-infectious diseases such as insulin resistance, EAE, and tissue repair. In these studies, IL-4Rα-dependent aaMφ have been shown to influence innate and adaptive immune responses with both beneficial or detrifmental outcomes, depending on the nature of the disease [6], [17], [18], [19], [20], [21], [22]. Arg-1 activation by IL-4Rα signalling is able to inhibit the activation of inducible nitric oxide synthase (iNOS) and therefore NO production, directly repressing classical macrophage activation. In a recent study, mice selectively lacking *Arg1* expression in macrophages during schistosomiasis were less susceptible than *LysM^cre^Il4ra^−/lox^* mice and developed higher Th2 cytokine responses and antigen-specific Th2 cell proliferation [23].

Besides inducing Arg-1, IL-4Rα signalling induces a large number of markers in macrophages [11], [24]. Of these, macrophage mannose receptor (MMR, *Mrc1*), “resistin-like” (Relm) (also referred as “found in inflammatory zone” (FIZZ)) molecules and chitinase-like molecules (also referred as “Ym”) have come to be considered as markers of the aaMφ phenotype [11], [25], [26]. MMR, a C-type lectin, is induced in granulomas during schistosomiasis and is involved in the uptake of soluble antigens by antigen presenting cells [25], [27], [28]. Resistin-like molecule alpha (*Retnla*/Relma/FIZZ1) upregulation has been recently shown to play essential roles in suppression of helminth-induced Th2 inflammation [29], [30]. Finally, though the chitinase-3-like 3 molecule (Ym1, *Chi3l3*) is strongly upregulated in macrophages during Th2-type immunity, its role remains unclear during helminth infection [31], [32]. It is also apparent that these markers can be upregulated independently of IL-4 and IL-13, with IL-10 in particular being able to induce the expression of MMR along with other factors including glucocorticoids [33], [34]. In addition, innate alternative activation of macrophages in response to chitin has also been reported [35]. Expression of these markers might therefore not be exclusively dependent on IL-4/IL-13 responsiveness of macrophages.

Although the absence of IL-4Rα-dependent aaMφ and T cell responses during schistosomiasis has been mainly studied at the systemic level [6], [10], little is known of their specific role(s) in the liver granuloma microenvironment. As the major alterations of the immune response depending on IL-4Rα might be restricted to the direct environment surrounding the parasite eggs, we focus in this study on the liver granuloma level to investigate how macrophage- or T cell-specific IL-4Rα responses affect the local responses directed against *Schistosoma* eggs. Our results demonstrate that the Th1/Th2 balance and the cellular composition of liver granulomas are not affected during infection of *LysM^cre^Il4ra^−/lox^* mice; but are both severely modified in *iLck^cre^Il4ra^−/lox^* mice developing granuloma cytokine and cellular responses very similar to the global *Il4ra^−/−^* mice. Although *LysM^cre^Il4ra^−/lox^* and *iLck^cre^Il4ra^−/lox^* granuloma macrophages show a global reduction of expression of aaMφ markers, we demonstrate that MMR and Ym1 protein expressions remain high in *LysM^cre^Il4ra^−/lox^* granuloma macrophages. This IL-4Rα-independent MMR^+^Ym1^+^ subpopulation was restricted at the periphery of *LysM^cre^Il4ra^−/lox^* granulomas, in contrast to *Il4ra^−/lox^* control mice where those cells were in close proximity to the parasite eggs. We finally used a parasite egg model with IL-10R blockade, and demonstrate that in absence of macrophage-specific IL-4Rα expression, IL-10 signalling can induce the expression of MMR and Ym1 in macrophages.

## Methods

### Mice

Macrophage/neutrophil-specific IL-4Rα-deficient mice (*LysM^cre^Il4ra^−/lox^*) and T cell specific IL-4Rα-deficient mice (*iLck^cre^Il4ra^−/lox^*) were generated with hemizygous *Il4ra^−/lox^* mice and homozygous *Il4ra^−/−^* mice were used as controls, as previously described [6], [10]. All mice used were on a BALB/c background. 8–12 week old sex-matched mice were obtained from the University of Cape Town specific-pathogen-free animal facility. All experiments were approved by the University of Cape Town Animal Ethics Committee.

### Parasite infections and antigen preparation

Mice were infected percutaneously with 100 cercariae of a Puerto Rican strain of *S. mansoni* obtained from infected *Biomphalaria glabrata* snails as described [10]. Snails and *S. mansoni* eggs isolated from livers of infected mice were obtained from Dr A.P. Mountford (University of York, UK). In some experiments, mice were injected intraperitoneally (i.p.) with 3,000 liver eggs in PBS. Soluble egg antigen (SEA) was prepared from purified eggs (liver) as described [36] and used at 20µg/ml.

### Liver granuloma-associated leukocyte purification

Granulomatous livers were finely cut in small pieces and digested in culture media containing 50µg/ml collagenase type IV (Sigma) at 37°C for 1.5h. Single cell suspensions were further passed through a 100-µm nylon mesh before leukocytes were isolated through a 30% Percoll cushion at 600×*g* during 15 min. Liver granuloma cells were further treated for 2 min with NH_4_Cl lysis buffer to lyse erythrocytes. Purified granuloma cells were used for *ex vivo* restimulation or flow cytometry analysis.

### Anti-IL-10 receptor treatment

Mice were inoculated intraperitoneally with PBS or PBS containing 4 µg anti-mouse IL-10Rα (R&D Systems, goat IgG) at day 0, 4 and 6 after injection of eggs.

### Antibodies and flow cytometry

mAbs targeting the following cell surface markers were used (the respective mAb clone names are given in italic): CD11b (APC-, FITC- or PE-conjugated, *M1/70*), I-A/I-E (MHC-II, FITC-conjugated or biotinylated, *M5/114*), F4/80 (PE-conjugated, *A3-1*, Caltag), Gr-1 (FITC- or PE-conjugated, *RB6-8C5*), Siglec-F (PE-conjugated, *E50-2440*), CD204 (Scavenger receptor-A, FITC-conjugated, *2F8*, Serotec), CD206 (MMR, PE-conjugated or biotinylated, *5D3*, Serotec), CD68 (macrosialin, FITC-conjugated, *FA-11*, Serotec), Dectin-1 (biotinylated, *2A11*, gift from Dr. G. Brown), CD80 (B7-1, PE-conjugated, *16-10A1*), CD86 (B7-2, PE-conjugated *GL1*), CD4 (PE-, FITC-conjugated, *GK1.5*, PerCP- conjugated, *RM4-5*), CD3 (PE- or FITC-conjugated, *145-2C11*), CD8 (FITC-conjugated or biotinylated, *53-6.7*), TCR_β_ (FITC-conjugated, *H57-597*), TCR_γδ_ (biotinylated, *GL3*), CD49b (pan-NK, biotinylated, *DX5*), CD19 (FITC-conjugated, *1D3*), CD124 (IL-4Rα, PE-conjugated or biotinylated, *M-1*). Staining specificity was verified with the appropriate isotype-matched antibody controls and compensation performed with single-stained samples before acquiring the multi-coloured samples. Incubations with antibodies were performed in washing buffer (PBS containing 0.1% BSA, 5mM EDTA and 2mM NaN_3_) supplemented with heat-inactivated rat serum (2%) and rat anti-mouse FcRII/III mAb (*2.4G2*, 10µg/ml). PerCP or APC-conjugated-streptavidin was used to detect biotinylated mAbs. For detection of intracellular Ym1 liver cells were fixed for 20 min on ice in 2% (wt/vol) paraformaldehyde and permeabilized during 30 min with 0.5% saponin buffer and further stained with biotinylated goat anti-Ym1/ECF-L (R&D systems). mAbs were from BD Pharmingen except where noted otherwise. Acquisition was performed using a FACSCalibur (BD Immunocytometry Systems), and data were analyzed by FlowJo software (Treestar). In some experiments, liver granuloma cells were sorted to high purity (≥98%) using a FACSVantage (BD Immunocytometry Systems) cell sorter. Sorted cell populations (SSC^high^CD11b^+^I-/I-E^−^F4/80^+/−^Gr-1^int/high^ granulocytes or CD11b^+^I-A/I-E^high^CD204^+^F4/80^+^ macrophages) were either used for RNA extraction or stained with Diff-Quick (Rapidiff set - Clinical Science Diagnostics) after cytospin for direct microscopic examination.

### *Ex vivo* restimulation and cytokine detection

Single-cell suspensions of mesenteric lymph node (mLN) cells or liver granuloma-associated leukocytes were cultured overnight in Iscove's modified Dulbecco medium (IMDM) containing 10% FCS, 2mM L-glutamine, 0.1 mM non essential amino acids, 1 mM Na Pyruvate (Invitrogen) and 50 µM 2-mercaptoethanol (Sigma) in presence or absence of 20 µg/ml SEA before cytokine detection by flow cytometry. IL-4, IL-10 and IFN-γ secreting cells were detected with the cytokine-specific mouse secretion assay detection kits (PE) (Miltenyi) according to the manufacturer's instructions. Dead cells were excluded from analysis by using 7-amino-actinomycin D (7-AAD, Sigma). For detection of intracellular IL-13, cells were further incubated 4h with monensin, fixed for 20 min on ice in 2% (wt/ol) paraformaldehyde and permeabilized during 30 min with 1× permeabilization buffer (eBioscience) before being stained with anti-IL-13-PE (eBioscience, *eBio13a*).

### Immunohistology

Liver tissue was embedded in OCT (Tissue-Tek, Sakura) before cryopreservation at −80°C. Seven-µm cryosections were cut and mounted onto 3-aminopropyltriethoxysilane (APES) coated slides, dehydrated overnight at 4°C before being fixed in ice-cold acetone. Washes were performed in 0.05% Tween-20 in PBS and staining steps in PBS containing 0.1% BSA. We used the primary antibodies to the following: CD206-Biotin (MMR, *5D3*, Serotec), Ym1/ECF-L-Biotin (goat IgG, R&D Systems), CD204-FITC (Scavenger receptor-A, *2F8*, Serotec), or iNOS (rabbit IgG, provided by Dr J.Pheilschifter, Germany). PE-conjugated streptavidin (BD Biosciences) was used to detect biotinylated primary antibodies after an avidin/biotin blocking step (Vector). FITC-conjugated goat anti-rabbit IgG secondary antibody (Abcam) was used for the detection of iNOS staining. Sections were washed in PBS before coverslipped in anti-fade fluorescent mounting medium (Dako). Staining specificity was verified by using irrelevant antibody controls. Images were taken with a Nikon Eclipse E400 microscope (Nikon), control by a NIS-Elements Basic Research 3.0 imaging system. Exposure times and fluorescence intensities were normalized to appropriate control images. Photomicrographs of liver granuloma focusing on autofluorescent parasite eggs were captured using a Nikon 5.0 mega pixels color digital camera (Digital SIGHT DS-SMc). Images were photographed separately for each fluorescent channels and were merged using Adobe Photoshop 7.0.

### Quantitative RT-PCR

Total RNA was extracted and purified with RNeasy Microprep kit (Qiagen) and cDNA synthesised with Transcriptor First Strand cDNA synthesis kit™ (Roche). Real-time PCR was performed using Lightcycler® FastStart DNA Master^PLUS^ SYBR Green I reaction mix (Roche) and the reactions run on a Lightcycler® carousel-based system (Roche). Primers used are listed in Table 1.

**Table 1:** List of gene-specific primer pairs used for quantitative real-time PCR.

Gene Product	Forward primer	Reverse primer
*Il4ra*	5′-TGACCTCACAGGAACCCAGGC-3′	5′-GAACAGGCAAAACAACGGGAT-3′
*Nos2*	5′-AGCTCCTCCCAGGACCACAC -3′	5′-ACGCTGAGTACCTCATTGGC -3′
*Arg1*	5′-CAGAAGAATGGAAGAGTCAG -3′	5′-CAGATATGCAGGGAGTCACC-3′
*Retnla*	5′-TCCCAGTGAATACTGATGAGA-3′	5′-CCACTCTGGATCTCCCAAGA-3′
*Mrc1*	5′-CTCGTGGATCTCCGTGACAC-3′	5′-GCAAATGGAGCCGTCTGTGC-3′
*Chi3l3*	5′-GGGCATACCTTTATCCTGAG-3′	5′-CCACTGAAGTCATCCATGTC–3′
*r12S*	5′-GGAAGGCATAGCTGCTGGAGGT-3′	5′-CGATGACATCCTTGGCCTGA-3′

### Statistical analysis

Statistical analyses were conducted using GraphPad Prism 4 software (www.graphpad.com). One-way ANOVA test was used to determine significant differences, with Bonferroni's multiple comparison post test applied to calculate significance values between samples.

## Results

### Characterization of macrophagic/granulocytic cell populations within liver granulomas in schistosomiasis

**Figure 1:**
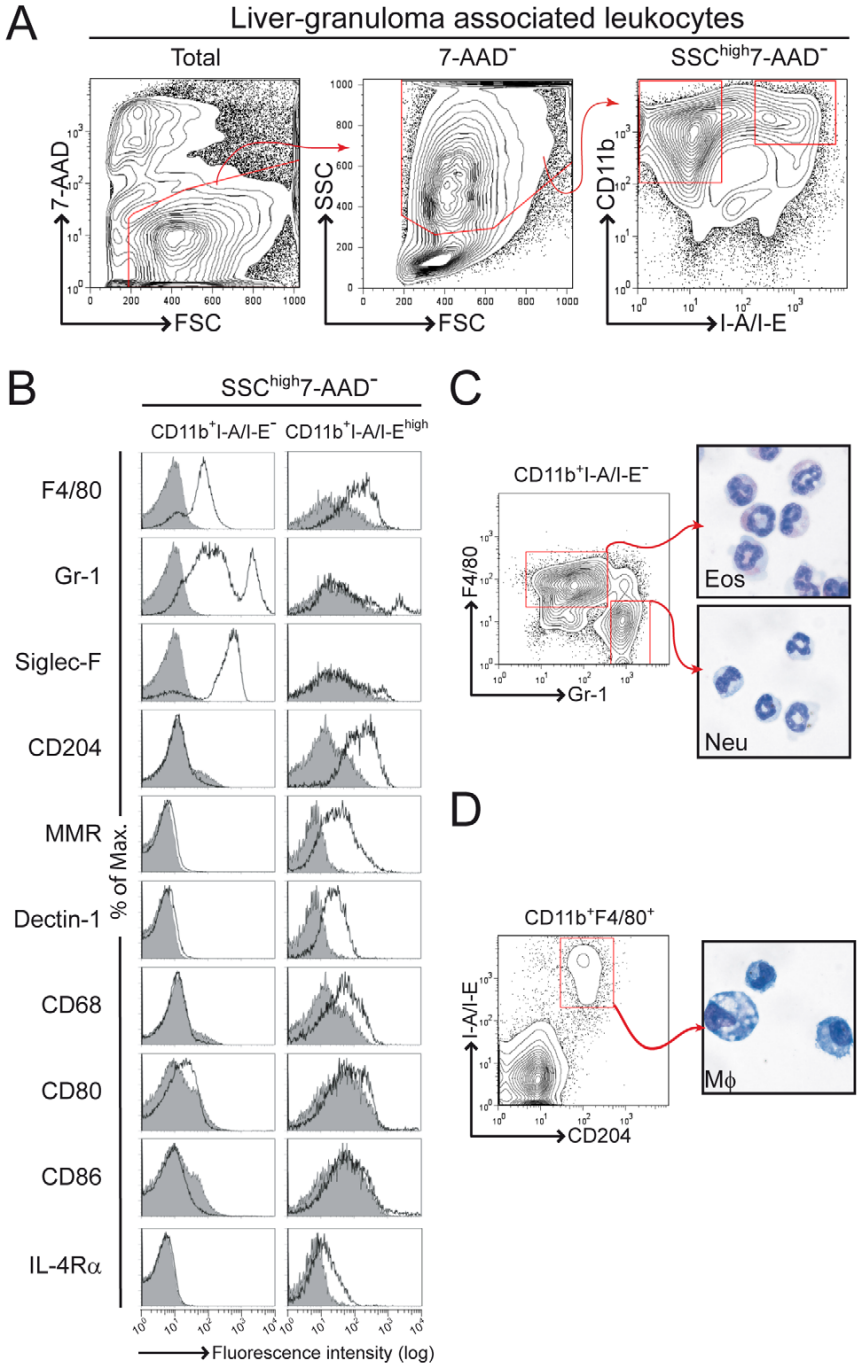
Flow cytometry analysis of liver granuloma during acute schistosomiasis. BALB/c mice were infected with 100 *S. mansoni* cercariae, killed 8 weeks later and liver granuloma-associated leukocytes isolated before 4-colour flow cytometry analysis. (A) Gating strategy for analysis of surface expression of CD11b and I-A/I-E on SSC^high^ and live (7-AAD^−^) liver granuloma-associated leukocytes. Outlined regions define CD11b^+^I-A/I-E^−^ and CD11b^+^I-A/I-E^high^, respectively. (B) CD11b^+^I-A/I-E^−^, and CD11b^+^I-A/I-E^high^ gated populations in A were analyzed for F4/80, Gr-1, Siglec-F, CD204, MMR, Dectin-1, CD68, CD80, CD86 and IL-4Rα expression, respectively. Greyscale histograms show relevant isotype control. (C) 4-colour flow cytometry analysis of liver granuloma-associated leukocytes gated on CD11b^+^I-A/I-E^−^ cells as described in A. F4/80 and Gr-1 double staining contour plot is shown. Outlined regions were sorted for cytospin and morphological analysis. (D) 4-colour flow cytometry analysis of liver granuloma-associated leukocytes gated on CD11b^+^F4/80^+^ cells as described in A. MHC-II (I-A/I-E) and scavenger receptor-A (CD204) double staining contour plot is shown. Outlined region was sorted for cytospin and morphological analysis. Data are representative of three independent experiments with similar results.

The major leukocytic cell types associated with the liver granuloma in *S. mansoni* infected wild-type mice are T cells, eosinophils and macrophages [3]. IL-4/IL-13-activated macrophages have been shown to play a crucial role in host survival during acute schistosomiasis [6]. Studies to define tissue macrophages are however complex as these cells can express different markers depending on the organ or tissue, in particular eosinophils are known to express high levels of both CD11b and F4/80 [37]. Here, we used a 4-colour flow cytometry strategy to detect and isolate liver granuloma-associated macrophages and granulocytes. Following exclusion of dead cells, a first gate was placed on CD11b^+^I-A/I-E^−^ cells for granulocytes. As opposed to inflammatory macrophages, Küpffer cells express no/low levels of CD11b [38], [39]. Therefore, a second gate was placed on CD11b^+^I-A/I-E^high^ for detection of granuloma macrophages (Fig. 1A). Further analysis of additional markers expression demonstrated that a large proportion of CD11b^+^I-A/I-E^−^ cells were also F4/80^+^, Gr-1^int^ or Gr-1^high^ (Ly-6G) and Siglec-F^+^ (sialic-acid binding Ig-like lectin, a CD33-related Siglec family member) (Fig. 1B). F4/80 and Siglec-F are both expressed on eosinophils but not on neutrophils [37], [38], [39], [40]. Most of the CD11b^+^I-A/I-E^−^ cells did not express macrophage-specific surface markers, such as macrophage scavenger receptor-A (CD204), MMR, Dectin-1 (a β-glucan receptor), macrosialin (CD68); nor did they express members of the B7 co-stimulatory molecules (CD80 and CD86) and expressed low levels of IL-4Rα (Fig. 1B). Subsequently, we identified a four colour staining with CD11b, I-A/I-E, F4/80, and Gr-1 as a clear definition and discrimination of CD11b^+^I-A/I-E^−^F4/80^+^Gr-1^int^ (and Siglec-F^+^) eosinophils and CD11b^+^I-A/I-E^−^F4/80^−^Gr-1^high^ (and Siglec-F^−^) neutrophils, confirmed by morphologic analysis of cytospins (Fig. 1C). Most of CD11b^+^I-A/I-E^high^ cells however did not express Siglec-F or Gr-1, but expressed F4/80, IL-4Rα and macrophage-specific markers, such as CD204, MMR, Dectin-1 and CD68 (Fig. 1B). Granuloma macrophages were therefore defined as CD11b^+^F4/80^+^I-A/I-E^high^CD204^+^ cells (with F4/80 not essential), confirmed by morphologic analysis of cytospins obtained from FACS-sorted CD11b^+^I-A/I-E^high^CD204^+^ cells (Fig. 1D). Thus, granuloma macrophages within the liver of *S. mansoni* infected mice are SSC^high^CD11b^+^I-A/I-E^high^CD204^+^.

### Cellular composition and cell-specific cytokine responses of liver granulomas in *LysM^cre^Il4ra^−/lox^* and *iLck^cre^Il4ra^−/lox^* mice

**Figure 2:**
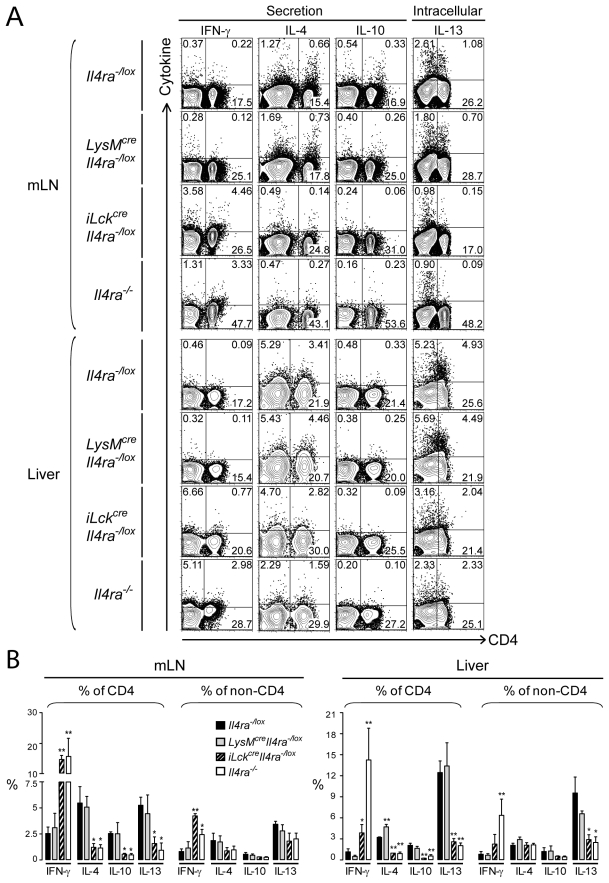
IL-4Rα-expressing macrophages do not affect cytokine responses in granulomas. *Il4ra^−/lox^*, *LysM^cre^Il4ra^−/lox^*, *iLck^cre^Il4ra^−/lox^* and *Il4ra^−/−^* mice were infected with 100 *S. mansoni* cercariae and killed 8 weeks later. Mesenteric lymph node (mLN) cells or liver granuloma-associated leukocytes (Liver) were then isolated and restimulated overnight with 20 µg/ml SEA before analyzed for *ex vivo* cytokine production capability. Staining of surface CD4 was followed by detection of IL-4, IL-10 and IFN-γ secretion in a cytokine catch assay or detection of intracellular levels of IL-13 after 4h of monensin treatment, as described in Methods. Live gate was placed on lymphocytes according to their forward- and side-scatter by flow cytometry. (A) Representative contour plots of IFN-γ, IL-4, IL-10 and IL-13-producing lymphocytes. Quadrant bars were set up on unstimulated cells and numbers represent percent cells in each quadrant. Data represent one out of three independent experiments with similar results. (B) Percentages of IFN-γ, IL-4, IL-10 or IL-13-producing cells were determined in gated CD4^+^ or non-CD4^+^ cell populations from mLN or liver granuloma-associated lymphocytes (Liver) (mean±SEM, *n* = 4). Data are representative of three independent experiments. *p<0.05; **p<0.001; ***p<0.001 compared to control *Il4ra^−/lox^* mice.

We previously reported that both *S. mansoni*-infected *LysM^cre^Il4ra^−/lox^* and *iLck^cre^Il4ra^−/lox^* mice developed hepatotoxicity and increased granuloma sizes [6], [10]. It is however unknown whether macrophage/neutrophil-specific or T cell-specific deletion of IL-4Rα affects the cellular composition or cytokine responses within liver granulomas. At 8 weeks post-infection (p.i.), corresponding to the peak of Th2 responses, leukocytes were isolated from granulomas of infected *Il4ra^−/lox^*, *LysM^cre^Il4ra^−/lox^*, *iLck^cre^Il4ra^−/lox^*,and global *Il4ra^−/−^* mice and analyzed by flow cytometry. *Il4ra^−/lox^* littermates were used as controls. Results obtained in Table 2 show that the relative frequencies of macrophages, neutrophils, eosinophils and lymphocyte subpopulations in granulomas were not affected by the specific impairment of IL-4Rα on macrophages, with eosinophils (CD11b^+^F4/80^+^Gr-1^int^Siglec-F^+^) dominating granulomatous cell composition in *LysM^cre^Il4ra^−/lox^* and infected *Il4ra^−/lox^* control mice. We observed however dramatically disrupted granuloma cell populations in *iLck^cre^Il4ra^−/lox^* mice with increased frequencies of macrophages (CD11b^+^I-A/I-E^high^CD204^+^), neutrophils (CD11b^+^F4/80^−^Gr-1^high^), and γδ-T cells. Though we previously showed that these mice developed bigger granulomas with reduced numbers of eosinophils per granuloma [10], *iLck^cre^Il4ra^−/lox^* mice showed high numbers of eosinophils in their liver (143.1×10^5^ cells in *iLck^cre^Il4ra^−/lox^* mice *vs.* 110.5×10^5^ cells in *Il4ra^−/lox^* mice). This apparent discrepancy could be explained by the fact that these mice had globally increased cell numbers per liver (data not shown). The cellular changes in *iLck^cre^Il4ra^−/lox^* mice were very similar to the disruption of the granuloma cell frequencies observed in *Il4ra^−/−^* mice, also showing increased CD4^+^ T cells and B cells. The disruption of the granulomas in *Il4ra^−/−^* mice could be explained by the 4-fold-reduced numbers of eosinophils (25.4×10^5^ cells in *Il4ra^−/−^* mice *vs.* 115.9×10^5^ and 110.6×10^5^ cells in *LysM^cre^Il4ra^−/lox^* and *Il4ra^−/lox^* mice, respectively) (Table 2). CD4^+^CD62L^high^ naive T cell numbers however changed with a 4-fold increase in infected *iLck^cre^Il4ra^−/lox^* mice and a 10-fold increase in infected *Il4ra^−/−^* mice compared to *Il4ra^−/lox^* control mice over-proportionally (28.3×10^4^ cells in *Il4ra^−/−^* mice and 11.9×10^4^ cells in *iLck^cre^Il4ra^−/lox^* mice *vs.* 4.3×10^4^ and 2.9×10^4^ cells in *LysM^cre^Il4ra^−/lox^* and *Il4ra^−/lox^* mice, respectively). In contrast, NK (CD11b^−^SSC^low^TCR_αβ_^−^CD49b^+^) and NK-T (CD11b^−^SSC^low^TCR_αβ_^+^CD49b^+^) cells showed significant lower frequencies in the liver of infected *Il4ra^−/−^* mice compared to *Il4ra^−/lox^* control mice (10.3% *vs* 17.3% and 8.2% *vs* 25.7%, respectively), resulting in a 2-fold cell number reduction of NK-T cells compared to control mice (2.9×10^5^ cells in *Il4ra^−/−^* mice *vs.* 5.8×10^5^ cells in *Il4ra^−/lox^* mice) (Table 2). Following *ex vivo* SEA restimulation of mesenteric lymph node (mLN) cells or liver granuloma-associated leukocytes, cytokine productions of CD4^+^ T cells and non-CD4^+^ cells were analyzed by multicolour flow cytometry, either by catch secretion assay (IFN-γ, IL-4, IL-10) or intracellular staining (IL-13) (Fig. 2). Cytokine catch secretion assays give information on cytokines secreted by a specific cell-type, gathering therefore advantages of both ELISA and intracellular staining. In both tissues, lymphocytes from *LysM^cre^Il4ra^−/lox^* mice produced similar levels of IL-13, IL-10 and IFN-γ cytokines compared to *Il4ra^−/lox^* control mice with slightly but significant higher levels of IL-4 in liver CD4^+^ cells compared to *Il4ra^−/lox^* control mice (Fig. 2B). In contrast, lymphocytes from both *iLck^cre^Il4ra^−/lox^* and *Il4ra^−/−^* mice produced less Th2 cytokines but more IFN-γ revealing a shift towards Th1-type responses within the granulomas, explaining the observed differences in cellular composition.

**Table 2:** Cellular composition of liver granulomas depending on macrophage/neutrophil or T cell-specific IL-4Rα deletion.

Gated population^a^	Marker (cell population)	Proportion of gated population^b^				Cell number per liver (×10^−5^)^c^			
		*Il4ra^−/lox^*	*LysM^cre^ Il4ra^−/lox^*	*iLck^cre^ IL4ra^−/lox^*	*Il4ra^−/−^*	*Il4ra^−/lox^*	*LysM^cre^ Il4ra^−/lox^*	*iLck^cre^ IL4ra^−/lox^*	*Il4ra^−/−^*
CD11b^+^SSC^high^	I-A/I-E^high^CD204^+^ (MΦ)	21.8±1.4	21.9±3.7	35.8±5.4^d^	39.2±3.4^d^	42.3±8.2	48.3±14.1	134.9±29.1^e^	65.9±22.7^d^
	F4/80^−^Gr-1^high^ (Neu)	14.0±4.6	16.6±2.9	24.2±1.8^d^	28.0±2.1^d^	27.3±5.3	36.6±10.6	91.2±19.7^e^	47.2±16.2
	F4/80^+^Gr-1^int^Siglec-F^+^ (Eos)	56.8±1.4	52.4±0.8	57.0±7.5	15.1±0.2^d^	110.5±21.3	115.9±33.6	143.1±30.9	25.3±8.7^e^
CD11b^−^SSC^low^	CD4^+^ (Th)	23.2±3.9	21.1±5.3	21.5±0.3	31.1±4.6^d^	19.5±3.8	16.3±4.7	27.7±6.0	32.5±11.2^d^
	CD8^+^ (CTL)	6.7±0.9	5.0±1.4	3.4±0.4^d^	6.3±1.4	5.6±1.1	3.9±1.1	4.4±0.9	6.5±2.3
	CD19^+^ (B)	18.7±0.1	21.2±0.4	15.1±0.2	33.0±9.8^d^	15.7±3.0	16.4±4.7	19.5±4.2	34.5±11.9^e^
CD11b^−^SSC^low^TCR_β_^−^	CD49b^+^ (NK)	17.3±4.0	13.2±0.4	16.9±1.0	10.3±0.3^d^	9.4±1.8	6.9±2.0	13.3±2.9^d^	7.4±2.6
CD11b^−^SSC^low^TCR_β_^+^	CD49b^+^ (NK-T)	25.7±2.5	23.3±6.8	15.4±2.9^d^	8.2±0.3^d^	5.8±1.1	4.5±1.3	4.7±1.0	2.9±1.0^d^
CD11b^−^SSC^low^CD4^+^	CD62_L_^high^ (Th naive)	1.5±0.2	2.5±0.2	4.0±0.1^d^	8.1±0.9^d^	0.3±0.1	0.4±0.1	1.2±0.3^d^	2.8±1.0^e^
	CD44^+^ (Th effector/memory)	73.2±5.4	73.2±8.2	69.8±5.6	63.0±7.9	14.3±2.8	12.7±3.7	21.0±4.5	22.0±7.5
CD11b^−^SSC^low^CD3^+^CD4^−^	TCR_γδ_^+^ (γδT)	5.0±1.4	6.7±3.0	10.4±3.9^d^	10.7±2.3^d^	0.5±0.1	0.5±0.2	1.5±0.3^d^	0.7±0.2

### Mannose receptor and Ym1 expression in liver granuloma-associated macrophages of *LysM^cre^Il4ra^−/lox^* mice

**Figure 3:**
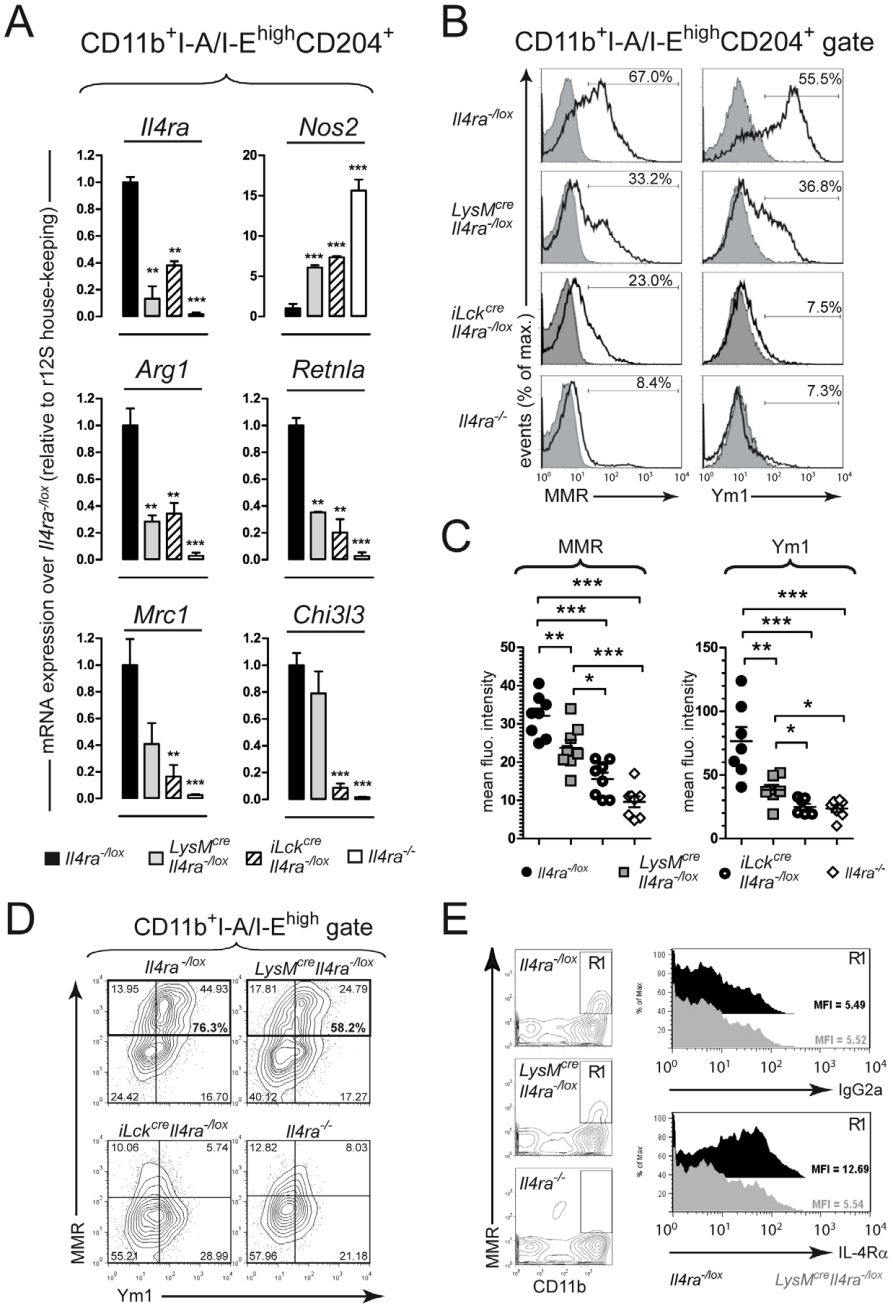
Mannose receptor and Ym1 expression in granuloma macrophages of *LysM^cre^Il4ra^−/lox^* mice. *Il4ra^−/lox^*, *LysM^cre^Il4ra^−/lox^*, *iLck^cre^Il4ra^−/lox^* and *Il4ra^−/−^* mice were infected with 100 *S. mansoni* cercariae, killed 8 weeks later and liver granuloma-associated leukocytes purified. (A) CD11b^+^I-A/I-E^high^CD204^+^ granuloma macrophages were sorted to high purity (>98%) and RNA extracted before analysis of expression levels of genes associated with macrophage activation by real time RT-PCR: IL-4Rα (*Il4ra*), nitric oxide synthase (*Nos2*), arginase 1 (*Arg1*), FIZZ1 (*Retnla*), macrophage mannose receptor (*Mrc1*), and Ym1 (*Chi3l3*). Relative expression levels normalized to r12S house-keeping gene are shown as fold-change over *Il4ra^−/lox^* mice values. Data is representative of two independent experiments with analysis of pooled samples (*n* = 4). Bars show mean±SD of analyses performed in triplicates. (B) 4-colour flow cytometry analysis of granuloma leukocytes gated on CD11b^+^I-A/I-E^high^CD204^+^ cells. Representative monoparametric histograms show stainings of surface macrophage mannose receptor (MMR) or intracellular Ym1 expression. Data is representative of four independent experiments of pooled samples (*n* = 4). Greyscale histograms show relevant isotype control. (C) Mean fluorescent intensities of MMR and Ym1 stainings based on analysis by flow cytometry as presented in B. Data show results of individual mice for 2 independent experiments. (D) 4-colour flow cytometry analysis of granuloma leukocytes gated on CD11b^+^I-A/I-E^high^ cells. Representative contour plots show MMR/Ym1 double staining and outlined regions show MMR-positive staining. Data is representative of two independent experiments of pooled samples (*n* = 4). (E) 4-colour flow cytometry analysis of granuloma leukocytes. Analysis gate (R1) was placed on SSC^high^CD11b^+^MMR^+^7-AAD^−^ and histograms show isotype IgG2a (upper panel) and anti-IL-4Rα (lower panel) stainings. Data is representative of two independent experiments of pooled samples (*n* = 4). Black, *Il4ra^−/lox^*; grey, *LysM^cre^Il4ra^−/lox^*. * *p*<0.05, **p<0.01; ***p<0.001.

We further compared gene expression levels of markers used to distinguish classical from alternative macrophage activation within liver granulomas during acute *S. mansoni* infection. Liver granuloma macrophages (SSC^high^CD11b^+^I-A/I-E^high^CD204^+^) were FACS-sorted to high-purity at 8 weeks p.i. (≥98%), RNA extraction performed, and gene expression profile analyzed (Fig. 3A). *Il4ra* (IL-4Rα) mRNA expression, observed in *Il4ra^−/lox^* control macrophages, was impaired in macrophages from *LysM^cre^Il4ra^−/lox^* mice and global *Il4ra^−/−^* mice, confirming the efficiency of *Cre*-*lox*P mediated DNA deletion in granuloma macrophages. Interestingly, *Il4ra* expression was also reduced in *iLck^cre^Il4ra^−/lox^* macrophages, suggesting a predominant classical activation in the granulomas of this mouse strain. Consistent with the IL-4/IL-13/IL-4Rα- and IFN-γ/IFN-γR-mediated macrophage activation by direct control of expression of the enzymes catalysing L-arginine, *LysM^cre^Il4ra^−/lox^*, *iLck^cre^Il4ra^−/lox^*, and global *Il4ra^−/−^* mice presented classically activated macrophages with heightened *Nos2* but reduced *Arg1* mRNA expression levels, compared to *Il4ra^−/lox^* control mice. This was in line with the expression pattern of the known alternative activation marker gene *Retnla* (FIZZ1), significantly reduced in macrophages from *LysM^cre^Il4ra^−/lox^*, *iLck^cre^Il4ra^−/lox^* and global *Il4ra^−/−^* mice, compared to *Il4ra^−/lox^* control mice. Of importance, the aaMφ marker *Chi3l3* and *Mrc1* gene expression levels were present although reduced for *Mrc1* in *LysM^cre^Il4ra^−/lox^* macrophages, resulting in Ym1 and MMR surface expression in about 30% of SSC^high^CD11b^+^I-A/I-E^high^CD204^+^ macrophages from *LysM^cre^Il4ra^−/lox^* mice. The expression of these two markers was however strongly reduced in both *iLck^cre^Il4ra^−/lox^* and global *Il4ra^−/−^* mice, compared to control *Il4ra^−/lox^* mice (Fig. 3B). Mean fluorescent intensities (MFI) values of MMR and Ym1 stainings were also significantly higher in macrophages from *LysM^cre^Il4ra^−/lox^* compared to both *iLck^cre^Il4ra^−/lox^* and global *Il4ra^−/−^* mice (Fig. 3C). The majority (76.3%) of MMR-expressing CD11b^+^I-A/I-E^high^ granuloma macrophages also expressed Ym1 in the *Il4ra^−/lox^* control mice (Fig. 3D). Absence of surface IL-4Rα expression was further confirmed by 4-colour staining on granuloma macrophages (Fig. 3E). MFI of IL-4Rα staining on gated CD11b^+^MMR^+^ cells revealed similar expression levels of the receptor between *LysM^cre^Il4ra^−/lox^* mice and isotype control (MFI = 5.54 and 5.52, respectively), whereas granuloma CD11b^+^MMR^+^ cells from *Il4ra^−/lox^* mice showed 2-fold increased expression of the IL-4Rα compared to *LysM^cre^Il4ra^−/lox^* mice and isotype control (MFI = 12.69) (Fig. 3E). Together, these data suggest that MMR and Ym1 can be expressed independently of macrophage-specific IL-4Rα signalling, whereas IL-4Rα-dependent T-cell responses are necessary for driving expression of aaMφ markers, including MMR and Ym1.

### Peripheral localisation of IL-4Rα-independent MMR- and Ym1-positive macrophages within egg-induced liver granulomas of *LysM^cre^Il4ra^−/lox^* mice

**Figure 4:**
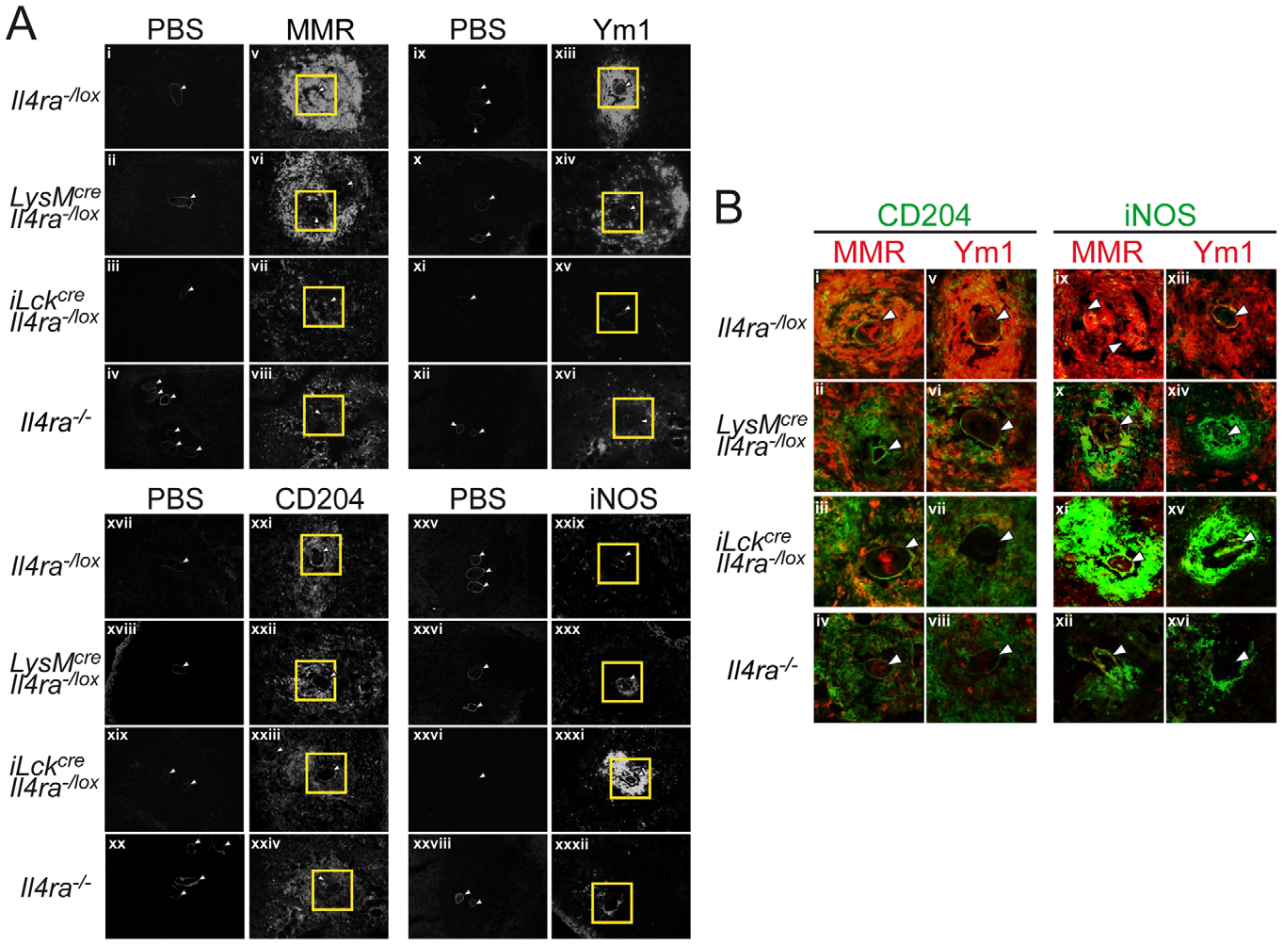
Localisation of mannose receptor and Ym1-expressing granuloma macrophages in close contact with *S. mansoni* eggs depends on their IL-4Rα signalling. Livers were collected at 8 weeks p.i. from *Il4ra^−/lox^*, *LysM^cre^Il4ra^−/lox^*, *iLck^cre^Il4ra^−/lox^* and *Il4ra^−/−^* mice and immunofluorescent stainings performed on cryosections. (A) Representative micrographs of MMR (panels v–viii), Ym1 (panels xiii–xvi), CD204 (panels xxi–xxiv) and iNOS (panels xxix–xxxii) stainings of liver cryosections as described in Methods. Stainings with secondary antibody (MMR, iNOS), streptavidin alone (Ym1) or isotype-control (CD204) is shown for each corresponding staining (MMR = i–iv, Ym1 = ix–xii, CD204 = xvii–xx, iNOS = xxv–xxviii). Note that MMR^+^ and Ym1^+^ cells are restricted to the periphery of *LysM^cre^Il4ra^−/lox^* granulomas. Outlined regions represent the areas magnified in B. (B) Representative micrographs of liver cryosections stained with scavenger receptor (CD204) for macrophages detection (panels i–viii, green) or iNOS for classically activated macrophages detection (panels ix–xvi, green); and co-stained for MMR (panels i–iv and ix–xii, red) or Ym1 (panels v–viii and xiii–xvi, red). Note the low frequency of CD204^+^ macrophages co-expressing MMR^+^ or Ym1^+^ cells around the parasite eggs (panels ii and vi) but the high levels of iNOS^+^ cells (panels x and xiv) in *LysM^cre^Il4ra^−/lox^* mice, suggesting these macrophages to be classically activated. White arrows indicate the parasite eggs. Original magnification: 400×. Data represent one of three independent experiments.

The results detailed in Figure 3 demonstrated that *LysM^cre^Il4ra^−/lox^* granuloma macrophages have retained expression of MMR and Ym1. This was surprising as we previously showed that liver granuloma from *LysM^cre^Il4ra^−/lox^* mice had low levels of MMR expression in close proximity to the eggs compared to *Il4ra^−/lox^* control mice [6]. To further determine the localisation of the MMR- and Ym1-positive macrophage subpopulation within the liver granuloma, immunofluorescent stainings were performed on liver cryosections at 8 weeks p.i. (Fig. 4). As expected from the expression data, MMR and Ym1 were highly expressed in granulomas from *Il4ra^−/lox^* control mice; and these cells co-expressing scavenger receptor A (CD204) were in close proximity to the parasite eggs in the centre of the granuloma (Fig. 4A and B). MMR- and Ym1-positive macrophages were restricted to the periphery of the granuloma in *LysM^cre^Il4ra^−/lox^* mice and nearly undetectable in both *iLck^cre^Il4ra^−/lox^* and *Il4ra^−/−^* mice. Instead, iNOS-positive, MMR- and Ym1-negative macrophages were in close proximity around the egg in the granuloma of *LysM^cre^Il4ra^−/lox^*,*iLck^cre^Il4ra^−/lox^* and *Il4ra^−/−^* mice (Fig. 4B), confirming previous observations [6], [10], [41]. These results show that a peripheral IL-4Rα-independent MMR- and Ym1-positive macrophage subpopulation surrounds the iNOS-producing classically activated macrophages present in close proximity to the egg within the liver granuloma of *LysM^cre^Il4ra^−/lox^* mice.

### IL-4Rα-independent MMR- and Ym1- positive macrophages depend on IL-10 signalling

**Figure 5:**
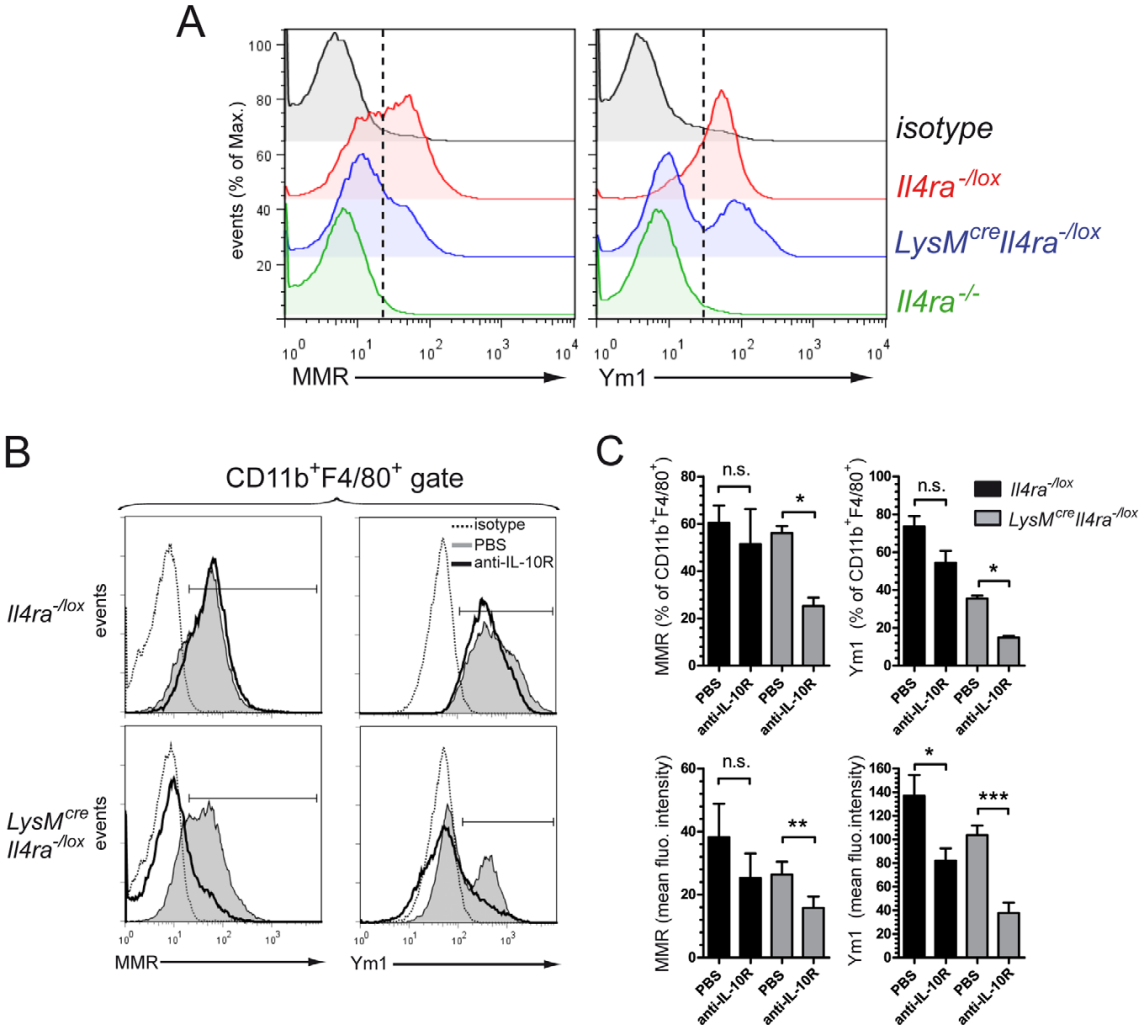
IL-10 signalling drives mannose receptor and Ym1 expression in macrophages independently of their IL-4Rα expression. Peritoneal cells were harvested 7 days after injection of 3000 *S. mansoni* purified eggs in the peritoneum of *Il4ra^−/lox^*, *LysM^cre^Il4ra^−/lox^*or *Il4ra^−/−^* mice. (A) Representative histograms of macrophage mannose receptor (MMR) or Ym1 expression by peritoneal macrophages gated on FSC^high^SSC^low^CD11b^+^F4/80^+^ cells after exclusion of peritoneal eosinophils (FSC^low^SSC^high^CD11b^+^F4/80^+^) [37]. Black, isotype control; red, *Il4ra^−/lox^*; blue, *LysM^cre^Il4ra^−/lox^*; green, *Il4ra^−/−^*. Broken lines show threshold for positive signal. Data are representative of two independent experiments of pooled samples (*n* = 3). (B) Anti-IL-10 receptor treatment. Four mice out of eight in each group received 4µg of anti-IL-10R i.p. at day 0, 4 and 6 post-injection. Overlays of representative monoparametric histograms of macrophage mannose receptor (MMR) or Ym1 expression by peritoneal macrophages are shown as described in A. Bracketed line indicates positive signal. Dotted line, isotype control; greyscale, untreated mice; bold line, mice treated with anti-IL-10R. (C) Percent and mean fluorescent intensities of peritoneal macrophages expressing MMR or Ym1 based on flow cytometry analysis in B. Data is representative of two independent experiments with mean±SEM (*n* = 3). *p<0.05, ** *p*<0.01, *** *p*<0.001.

IL-10 signalling can induce MMR expression in macrophages [34]. We therefore investigated whether IL-10 could drive the MMR and Ym1 expression in macrophages of *LysM^cre^Il4ra^−/lox^* mice. To test this hypothesis we used a local egg model by injecting purified *S. mansoni* eggs intraperitoneally to induce Th2 responses and elicit macrophage activation in *Il4ra^−/lox^*, *LysM^cre^Il4ra^−/lox^* and *Il4ra^−/−^* mice. At 7 days p.i., corresponding to the peak of Th2 responses [42], MMR and Ym1 were highly expressed in peritoneal macrophages of *Il4ra^−/lox^* control mice (Fig. 5A). To investigate peritoneal macrophages, we placed a live gate on FSC^high^SSC^low^CD11b^+^F4/80^+^ cells. Here the association of the CD11b, F4/80 markers and side/forward scatter allows discrimination between eosinophils and macrophages, as previously described [37]. Similar to that observed in macrophages from liver granuloma, a subpopulation of cells expressing MMR and Ym1 was present in peritoneal macrophages of *LysM^cre^Il4ra^−/lox^* mice but absent in peritoneal macrophages from *Il4ra^−/−^* mice (Fig. 5A). *In vivo* blockade of IL-10 signalling *via* anti-IL-10 receptor antibody treatment was sufficient to significantly reduce the protein expression of both MMR and Ym1 in peritoneal macrophages of *LysM^cre^Il4ra^−/lox^* mice (Fig. 5B and C). These results demonstrate that IL-10 drives expression of MMR and Ym1 in macrophages independently of macrophage IL-4Rα.

## Discussion

Recent *S. mansoni* infection studies demonstrated that *LysM^cre^Il4ra^−/lox^* mice, deficient for IL-4Rα-responsive aaMφ succumb to acute *S. mansoni* infection, due to the development of egg-induced gut inflammation, severe liver damage, and despite the formation of egg-induced granulomas and unaffected fibrosis in the liver [6]. These observations were very similar to the pathology developed by infected global *Il4ra^−/−^* mice [6], or *Il4^−/−^* and *Il4^−/−^Il13^−/−^* mice which developed hepatotoxicity and endotoxaemia due to the degradation of the intestinal barrier [5], [9]. Antibiotic treatment in infected *LysM^cre^Il4ra^−/lox^* mice resulted only in partial protection [6], suggesting that in addition to gut inflammation and systemic leakage of the intestinal content, hepatotoxic lesions could also explain the increased mortality. In addition to these findings, we also recently demonstrated, using *iLck^cre^Il4ra^−/lox^* mice, that IL-4Rα responsiveness by T cells was required for host survival mainly for controlling liver damage caused by the parasites eggs [10]. As *iLck^cre^Il4ra^−/lox^* mice did not develop gut injury or endotoxaemia, we concluded that IL-4Rα responsiveness by T cells is not essential for the control of intestine-derived sepsis. In this study we focused on the liver granuloma microenvironment and the consequences of impaired IL-4Rα signalling in macrophages or T cells on the local cellular responses directed against the parasite eggs.

Granulomas induced by *S. mansoni* contain both myeloid cells (mainly eosinophils and macrophages) and lymphoid cells (mainly T cells) [3]. Several markers are readily available for staining murine tissue macrophages but their expression can be highly tissue-dependent [43]. Most of the studies using such markers have been performed with blood, spleen or bone-marrow but little is known on granuloma macrophage markers during schistosomiasis. Liver granulomas are tightly organized, which renders their disruption difficult without affecting cell viability, especially macrophages. Though collagenase can in some conditions cleave surface proteins, comparison between collagenase-treated or untreated organs gave similar results concerning the expression levels of standard surface markers (including CD11b, F4/80, CD11c, Gr-1, and I-A/I-E), suggesting that collagenase treatment had no major influence on surface marker detection (data not shown). The combination of CD11b and/or F4/80 and MHC-II (I-A/I-E) was sufficient to specifically detect and isolate macrophages from granulomas. These macrophages were defined as SSC^high^CD11b^+^(F4/80^+^)I-A/I-E^high^CD204^+^ cells, showed a macrophage-like morphology and expressed high levels of macrophage-specific markers such as scavenger receptor-A (CD204), mannose receptor (MMR), β-glucan receptor (Dectin-1) and macrosialin (CD68). In parallel, we defined granuloma eosinophils as SSC^high^CD11b^+^F4/80^+^I-A/I-E^−^Gr-1^int^Siglec-F^+^ and neutrophils as SSC^high^CD11b^+^F4/80^−^I-A/I-E^−^Gr-1^high^Siglec-F^−^.

Despite increased liver granuloma sizes, previously reported in infected *LysM^cre^Il4ra^−/lox^* mice [6], neither the cellular composition nor the Th1/Th2 cytokine profiles were affected. In contrast, we observed severe changes in granuloma cell composition and morphology of infected *iLck^cre^Il4ra^−/lox^* and *Il4ra^−/−^* mice. Interestingly, a recent report showed that infected *LysM^cre^Arg1^−/lox^* or *Tie2^cre^Arg1^lox/lox^* mice (deficient for Arg1-expressing macrophages) did not develop lethal endotoxaemia or hepatotoxicity but showed increased Th2 responses and increased chronic disease [23]. Further, Arg1-expressing aaMφ were able to suppress schistosome-specific T cell proliferation, suggesting a possible mechanism for downmodulating granulomatous inflammation and slow the progression of Th2-driven fibrosis during chronic infection [23]. Similarly, studies in *Retnla*-deficient mice showed that absence of resistin-like molecule alpha (*Retnla*/FIZZ1) exacerbated Th2 cytokine responses in *S. mansoni*-egg-induced lung inflammation, suggesting that *Retnla*/FIZZ1 is able to control effector Th2 responses in *S. mansoni* egg-induced inflammation and infection [29], [30]. As IL-4Rα-sinalling is upstream from the induction of *Arg1* and *Retnla* in aaMφ, it might be not surprising that *LysM^cre^Il4ra^−/lox^* mice have a more severe disease phenotype already detrimental in the acute phase. The relative normal granuloma morphology, cellular composition and Th1/Th2 cytokine responses in the liver of *LysM^cre^Il4ra^−/lox^* mice but more severe liver pathology in *iLck^cre^Il4ra^−/lox^* mice, suggest that abrogation of IL-4-promoted Th2 responses in combination with impairment of aaMφ negatively affect the control of liver pathology in mice. Together with the chronic phenotype and aaMφ-mediated T cell suppression observed in *LysM^cre^Arg1^−/lox^* mice, aaMφ seem to control T helper cell responses in an optimal balance. Future studies need to further investigate the molecular mechanisms of how and which T cell subpopulations are controlled by aaMφ.

Interestingly, *LysM^cre^Il4ra^−/lox^* liver granulomas retained expression of MMR and Ym1 in a subpopulation of macrophages, confirmed in mRNA and protein (*Chi3l3*) or protein (*Mrc1*) expression analyses. Interestingly, MMR^+^Ym1^+^ macrophages were not observed in *iLck^cre^Il4ra^−/lox^* mice, suggesting that IL-4Rα-dependent Th2 responses drive MMR and Ym1 expression independently of IL-4Rα-responsive macrophages in *LysM^cre^Il4ra^−/lox^* mice. After staining of scavenger receptor-A (CD204) *in situ* to specifically detect macrophages [44], [45], we showed that macrophages were in close proximity to the parasite eggs within the granuloma in all mouse strains. However, macrophages expressing MMR or Ym1 were only found at the periphery of the *LysM^cre^Il4ra^−/lox^* granulomas, while present in close proximity to the eggs in the *Il4ra^−/lox^* control granulomas. As previously demonstrated, MMR- or Ym1-positive cells were absent of global *Il4ra^−/−^* granulomas [25] and we observed nearly undetectable MMR^+^ or Ym1^+^ cells in *iLck^cre^Il4ra^−/lox^* granulomas. Instead classically activated macrophages, defined by their iNOS expression were centred on the eggs in *LysM^cre^Il4ra^−/lox^*, *iLck^cre^Il4ra^−/lox^* and global *Il4ra^−/−^* granulomas (Fig. 4B). Increased iNOS activity in these mice strains could explain the heightened hepatocellular damage observed after *S. mansoni* infection [6], [10]. Together, these results suggest that IL-4Rα-responsiveness in aaMφ regulates their ability to migrate in close proximity and/or interact with the parasite eggs. The factors that could be responsible for the interaction with the eggs are not known, but a possible candidate could be MMR. This receptor encodes a C-type lectin, which has been involved in antigen uptake by antigen-presenting cells and components of *S. mansoni* eggs as well as a fraction of their egg-secreted molecules are ligands of MMR [25]. Furthermore, a study demonstrated that SEA can be internalized by DCs through the C-type lectins dendritic cell specific ICAM-3 grabbing non integrin (DC-SIGN), macrophage galactose-type lectin (MGL) and MMR [28]. We did not look at the expression of other C-type lectins, but the presence of macrophages expressing MMR in the proximity of the parasite eggs could allow IL-4Rα-responsive aaMφ to interact with antigen-specific T cells and also play regulatory functions on effector T cells as previously suggested [6], [23], [29], [30], [46]. Although Ym1 was described as a chitinase-like secreted protein, it lacks any chitinase activity and *S. mansoni* eggs do not contain chitin [47], [48]. A recent study described Ym1 to digest glycosaminoglycans and might therefore play a role in egg killing and/or antigen processing [49]. It has previously been suggested that Ym1 could encapsulate pathogens [31] and our observation of Ym1-expressing macrophages in the proximity of the parasite eggs supports this hypothesis. Encapsulation of the parasite eggs may serve to protect the host from pro-inflammatory molecules and harmful factors released by the eggs. Macrophages isolated from granulomas or macrophages elicited with *S. mansoni* egg components have previously been proposed to act as “myeloid suppressive cells (MSCs) by inducing clonal anergy in egg-antigen-specific Th1 cells by unknown mechanisms [46], [50], [51], [52], [53], [54]. Although we did not detect changes in cellular composition or cytokine production in the liver granulomas of *LysM^cre^Il4ra^−/lox^* mice, these mice quickly die from increased systemic Th1-type responses and acute infection due to uncontrolled gut inflammation [6]. Recently established MMR-deficient mice [55], generation of Ym1-deficient mice should allow further studies to address the role of such aaMφ biomarkers.

Previous studies proposed that MMR and Ym1 expression by macrophages could directly be driven by bacterial endotoxin or chitin [35], and independently of IL-4Rα signalling [56]. Linehan and colleagues (2003) previously described a complete absence of MMR expression in the liver granulomas of global *Il4ra^−/−^* mice [25], demonstrating that IL-4Rα responsiveness is essential for MMR expression in macrophages recruited to the granulomas. These authors however described resident liver macrophages to retain MMR expression and we also observed MMR expression in resident Küpffer cells (Fig. 4A). As Küpffer cells express no/low levels of CD11b in contrast to inflammatory macrophages [38], [39], we can exclude any contamination with these cells in our gating strategy targeting SSC^high^CD11b^+^(F4/80^+^)I-A/I-E^high^CD204^+^ granuloma macrophages.

Our findings demonstrate that direct IL-4Rα signalling in a subpopulation of granuloma macrophages is not essential for MMR and Ym1 expression and these cells are restricted to the periphery of *LysM^cre^Il4ra^−/lox^* granulomas. IL-10 signalling in macrophages can induce MMR expression [34] and Ym1 expression is downregulated in *Il10^−/−^* and *Il10^−/−^Il4^−/−^* deficient mice [32]. These observations, together with reduced IL-10 production and impaired aaMφ markers (including MMR and Ym1) in *iLck^cre^Il4ra^−/lox^* and *Il4ra^−/−^* liver granulomas, prompted us to consider IL-10 as a good candidate to explain the IL-4Rα-independent expression of MMR and/or Ym1 in macrophages. In order to focus on the egg-induced inflammation in the *LysM^cre^Il4ra^−/lox^* mice, we chose to use an egg model to induce acute Th2 responses following challenge in the peritoneum. Here, we showed evidence by *in vivo* IL-10R signalling blockade that IL-10 signalling was responsible for the retained expression of MMR and Ym1 expression in *LysM^cre^Il4ra^−/lox^* peritoneal macrophages following *S. mansoni* egg exposure and induction of Th2 cytokine responses, while no significant effect could be observed in macrophages of *Il4ra^−/lox^* control mice. Although elicitation of macrophages in the peritoneal cavity by *S. mansoni* eggs might not exactly reflect what occurs in the liver granuloma during live infection, data generated with peritoneal macrophages gave similar results as those obtained with granuloma macrophages. Control *Il4ra^−/lox^* mice over-expressed both MMR and Ym1, whereas expression of those markers in peritoneal macrophages from *Il4ra^−/−^* mice was strongly impaired. Moreover, *LysM^cre^Il4ra^−/lox^* peritoneal macrophages also retained expression of MMR and Ym1 after injection of eggs and anti-IL-10R treatment blocked the expression of both markers. These results indicate that IL-10 plays a key role in the retained expression of MMR and Ym1 in the liver granulomas of *LysM^cre^Il4ra^−/lox^* mice. The macrophages retaining expression of MMR and Ym1 in infected *LysM^cre^Il4ra^−/lox^* mice could therefore represent a cellular subpopulation with IL-4Rα-independent aaMφ-related regulatory activities, driven by IL-10. The presence of such macrophage population could explain why *LysM^cre^Il4ra^−/lox^* liver granulomas do not have disrupted cellular or cytokine responses, in contrast to mice having impaired Th2 responses and showing strongly reduced IL-10 production such as *iLck^cre^Il4ra^−/lox^* and *Il4ra^−/−^* mice. Supporting these results, Herbert *et al.* recently showed that *in vivo* IL-10R neutralization results in increased hepatocellular damage without affecting gut inflammation in schistosomiasis [57]. Hepatotoxicity was however observed in infected *LysM^cre^Il4ra^−/lox^* mice [6], suggesting that IL-10 without IL-4/IL-13 signalling in macrophages might not be sufficient to control liver tissue damage. Future work on IL-4Rα-independent MMR^+^Ym1^+^ macrophages from liver granulomas may define whether those cells have an “alternative” or “deactivated” phenotype, which was previously described to be mediated by IL-10 signalling [11].

In conclusion, we demonstrated that IL-4Rα-responsive macrophages are dispensable for the control of cellular and cytokine responses in liver granulomas during acute schistosomiasis, whereas IL-4Rα-responsive T cells are necessary to sustain cellular composition and morphology of the liver granuloma. Though our results suggest that macrophage-specific IL-4Rα expression is necessary for an adequate interaction between aaMφ and the parasite eggs, we showed that MMR and Ym1 expression may be induced *via* IL-10R signalling in absence of IL-4Rα signalling in macrophages. Taken together, our results suggest that IL-4Rα-derived Th2 cytokine responses are essential to drive aaMφ in the liver and demonstrate that in absence of macrophage-specific IL-4Rα, IL-10 signalling induces MMR^+^Ym1^+^ macrophages. Future investigation may clarify whether IL-10-driven IL-4Rα-independent MMR^+^Ym1^+^ macrophages have important functions in the control of liver granulomatous inflammation.
